# Psychiatric, cognitive functioning and socio-cultural views of menstrual psychosis in Oman: an idiographic approach

**DOI:** 10.1186/s12905-020-01060-z

**Published:** 2020-09-29

**Authors:** Nasser Al-Sibani, Mandhar Al-Maqbali, Sangeetha Mahadevan, Salim Al-Huseini, Muna Al-Muzeni, Samir Al-Adawi

**Affiliations:** 1 Department of Behavioral Medicine, College of Medicine & Health Sciences, Sultan Qaboos University, P.O. Box 35, P.C. 123, Al Khoudh, Muscat, Sultanate of Oman; 2 Psychiatry Residency Training Program, Oman Medical Specialty Board, Muscat, Oman

**Keywords:** Psychotic disorders, Menstrual cycle, Neurocognition, Transcultural aspects

## Abstract

**Background:**

Most documented cases of menstrual psychosis have been from Euro-American populations with reports from cross-cultural populations being only a few. A primary aim was to determine whether the cyclical/episodic nature of menstrual psychosis among case series observed at a tertiary care unit in Oman fulfills the diagnostic criteria of *the International Classification of Diseases* (ICD-10) and diverge into Brockington’s sub-types (World Psychiatry. 2005;4(1):9–17). Related aims were to solicit measures of psychometric functioning of those with menstrual psychosis and associated idioms of distress*.*

**Methods:**

A series of consecutive patients seeking psychiatric consultation from January 2016 to December 2017 were screened via structured interview—Composite-International Diagnostic Interview (CIDI) and Brockington’s sub-types. The identified patients (*n* = 4) also underwent psychometric evaluation including examination of affective functioning, intellectual capacity and neuropsychological functioning (i.e.attention and concentration, learning and remembering, executive function, processing speed and speech and language). The analysis of outcome measures was via an idiographic approach.

**Results:**

The spectrum of distress among people with menstrual psychosis does not fit existing psychiatric nosology. Evaluations revealed that a majority of the participants displayed something akin to morbid phenomena relating to manic and psychotic symptoms. In the parlance of traditional Omani society, this would be termed “spirit possession”. In terms of classification by timing within the menstrual cycle as expounded by Brockington, the present case series in Oman fulfilled the definition of catamenial psychosis and paramenstrual psychosis. With regard to psychometric function, all participants performed adequately on indices of intellectual functioning but appeared to have impairments in neuropsychological functioning, including the dimensions of processing speed, episodic memory, and executive functioning. Within the given society, the periodicity of mind alteration has been attributed to spirit possession.

**Conclusions:**

This is one of the first case series of its kind in the country elucidating whether the manifestation of menstrual psychosis among individuals in Oman fulfills the subtypes postulated by Brockington. The present case series suggests that menstrual psychosis is marked with neuropsychological impairments that were previously observed in other phasic manic episodes or brief psychotic disorders.

## Introduction

*Psychosis menstrualis* or menstrual psychosis had first featured in a monograph entitled “psychosis menstrualis” by Krafft-Ebing. He has been credited for coming up with the first description and classification of menstrual psychosis [1, 2]. More recently, menstrual psychosis has attracted the attention of other investigators too, most notably through the work of Brockington [1]. Much (if not all) of the work in menstrual psychosis has been in the form of case studies taking a phenomenological approach.

Menstrual psychosis is characterized by acute onset of symptoms akin to psychotic-manic symptoms and manifests as problems of either externalization, internalization or both. According to Brockington [1], these symptoms show a relapsing-and-remitting pattern in sync with the menstrual cycle [1, 3]. Within the background of existing literature, Brockington [1, 4] has reviewed case studies and postulated that there are several subtypes of menstrual psychosis including catamenial psychosis, paramenstrual psychosis, mid-cycle psychosis and epochal menstrual psychosis.

Menstrual psychosis is not considered a “specific disease entity” in current nosology. However, the temporal relationship between the onset and remission of symptoms specific to stages of the menstrual cycle has prompted many experts to regard menstrual psychosis as a ‘unique disorder’ [5]. The emergence of psychotic-manic-like symptoms appears to be orthogonal to menstruation-associated distress such as premenstrual tension, premenstrual syndrome, premenstrual depression or dysphoric disorder or menstrual mood disorder [6].

Many case reports have emerged from different parts of the world [3] but the documentation of menstrual psychosis in the Arabian Gulf is yet to happen. In the following study, we described a case series of menstrual psychosis in Oman. Rather than simply exploring the phenomenology of menstrual psychosis, it is increasingly recognized that cognitive deficits (neuropsychological and intellectual functioning) are likely to play a central role in the severity, disability and prognosis, and are likely to shape the quality of life among people with psychotic illnesses [7, 8]. As previous reports have only documented the clinical features of menstrual psychosis, this study examines ‘higher functioning’ including intellectual and cognitive functioning that we were able to tap into using conventional, intellectual and neuropsychological batteries.

The incidence of menstrual psychosis in societies in transition such as Oman and previously in India [5], has been instrumental in discounting the view that menstrual psychosis might be a culture-bound syndrome exclusive to the industrialized countries of Western Europe, North America and the Asia Pacific region [4, 9–12].

## Methods

### Setting

This is a descriptive study of a series of consecutive patients seeking psychiatric consultation from tertiary care (from January 2016 to December 2017) at the Department of Behavioral Medicine & Psychiatry, Sultan Qaboos University Hospital. The Department offers both in-patient and out-patient services. Oman, a country of approximately 2.5 million people [13], has a universal free healthcare system compartmentalized into primary, secondary and tertiary care.

### Psychometric evaluation

#### Structured interview

The semi-structured interview was derived from the Composite International Diagnostic Interview (CIDI) [14]. The CIDI has been successfully employed among many linguistic and cultural groups around the world, including Omanis [15]. The interview was conducted by psychiatrists who were well versed in its use and blinded to the results of intellectual and neuropsychological evaluation. A diagnosis was made after deliberation among authors according to the criteria of the ICD-10. In case the client did not fulfill the criteria outlined in CIDI, the clinical team deliberated whether the core symptoms could parallel those featured in the World Health Organization’s International Classification of Diseases and Health-Related Problems, Tenth Revision (ICD-10) [16]. In addition to CIDI, the cases were also labeled according to the fulfillment of various subtypes of menstrual psychosis using their psychosocial history, as postulated by Brockington (premenstrual psychosis, catamenial psychosis, paramenstrual psychosis, mid-cycle psychosis and epochal menstrual psychosis) [1].

#### Affective functioning

Mood status was solicited via the ***Calgary Depression Scale for Schizophrenia*** (CDSS) [17]. The CDSS is a 9-item scale which has been shown to tap into depressive symptoms of people with psychotic illnesses. It has been established to have adequate psychometric properties across various ethnic and linguistic groups around the world [18, 19] including the Arabic version that is currently being used [20]. The CDSS is scored as follows: 0–9 = no depression, scores 10–15 = mild depression, scores 16–23 = moderate depression, scores > 24 = serious depression.

#### Intellectual capacity/reasoning ability

We employed Raven’s Progressive Matrices (RPM) to gauge participants’ analytic intelligence or non-verbal reasoning ability [21]. For brevity, this study reported intelligence quotient (IQ) in percentile scores. Being a non-verbal IQ test, RPM has been reported to have heuristic value in cross-cultural settings including an Arabic-speaking population [22].

#### Neuropsychological functioning

We assessed different cognitive domains of attention and concentration (Digit Span) [23], learning and remembering (California Verbal Learning Test) [24], executive function (Wisconsin Card Sorting Test [25], processing speed (Digit Symbol) [26] and speech and language [27]. For brevity, performance on each test has been expressed in percentile scores as detailed elsewhere [28] based on normative data from Omani populations [25, 27].

## Results

### Case report 1 (AA): premenstrual psychosis

AA is a 23-year old single Omani woman. She was brought by her parent to the psychiatric clinic with a 3-year history of the cyclical presentation of a short episode of clouded sensorium and abrupt onset of psychosis. Her thoughts and emotions had been impaired to the extent that her family had begun to believe that she had been ‘possessed’ by a malevolent spirit. Her mother noted that her distress occurred during the second half of her menstrual cycle and ended at the onset of menstrual bleeding. During the second half of her menstrual cycle, AA was reported as showing symptoms of dysphoric mania, isolating herself and crying unremittingly without substantive reason. The episodes of negativistic behavior tended to be superseded by a state of overactivity and euphoria characterized by grandiose beliefs, inappropriate irritability and social behavior and increased talking speed or volume. AA’s symptoms were reported to recede upon the onset of menstrual bleeding. While sedulous dating was not feasible, she was described to have had approximately 6–8 episodes of ‘possession’ in a year, with a regular occurrence every month.

She had no relevant medical history and denied having a family background of persistent mental disorders. She also denied having consumed any mind-altering substances including tobacco or its rejuvenated forms and alcohol. A routine urine drug screening did not reveal the presence of any illicit drugs in her system.

Premorbid, AA met her developmental milestones without difficulty. AA had her first menstrual cycle at the age of 13 with regular cycles. She acquired 12 years of formal education and graduated with a secondary school leaving certificate with average performance. But due to her recurring distress, she did not seek further education or employment.

During her consultation in our unit (taking place after the onset of menstrual bleeding), the clinical team noted that she was asymptomatic, interactive and socially active. She informed the clinical team that she tended to have ‘strange feelings’ and ‘weird experiences’ starting during the second half of her menstrual cycle. Overall, she expressed foggy awareness of her recurring distress.

**Fig. 1:**
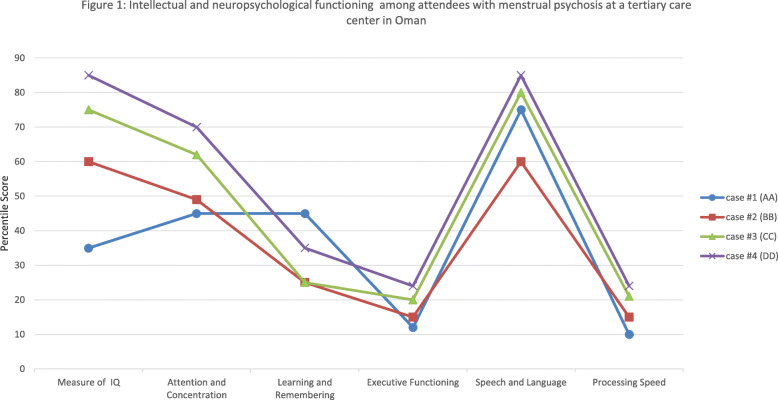
Intellectual and neuropsychological functioning among attendees with menstrual psychosis at a tertiary care center in Oman.

Physical examination was unremarkable and her medical workup—hormonal study, brain computerized tomography (CT) scan, and electroencephalogram (EEG)—was inconclusive. She was also subjected to neuropsychological testing and evaluation of mood (see Fig. 1). AA scored 23 on the ***Calgary Depression Scale for Schizophrenia*** [17]. Such a score implied the presence of moderate depressive symptoms.

Olanzapine (5 mg)—an antipsychotic—was instituted once every day to which she was deemed to be compliant. The clinical team noted that the severity of her distress significantly receded but still manifested in episodes of screaming and lability and, as often the case, dissipated upon the start of menstrual bleeding. As the symptoms appeared to be atypical to those featured in CIDI, the clinical team suggested the tentative diagnosis of a manic episode, unspecified (F309)/brief psychotic disorder (F24). During the subsequent follow-up, her antipsychotic, olanzapine, was tapered up to 10 mg once daily and it was noted that the patterns of her symptoms appeared to generally be aligned with her menstrual cycle.

During subsequent follow-up (6 months later), it appeared that her distress was exacerbated after approximately 28 days. Overall, her episodes of manic and psychotic-like symptoms remained the same but significantly reduced in terms of duration and veracity of distress.

### Case report 2 (BB): Catamenial psychosis

BB is a 34-year-old married woman with 3 children from the urban part of Oman. She approached the unit at the present hospital with her husband and her female siblings who helped provide us with anamnestic data. According to them, BB went through clouded sensorium which lasted for a short duration. The distress was accompanied by abrupt onset of inappropriate emotions, trouble in concentrating, suspiciousness, and spending a lot more time alone than usual; episodes that tended to reappear monthly throughout her adult life. The family attributed her distress to ethnopathology. She had been taken to a traditional healer who recommended dietary changes, herbal medicine and attributed her behavior to an ‘invading evil spirit’.

During these episodes, she had had poor self-care, hygiene, oral intake and a disturbed sleep pattern. She had once woken up at midnight to sprinkle her children with water. During exacerbation of her distress, she became suspicious of her husband and the female domestic servant. She displayed odd mannerisms and stereotypical behavior along with rituals of cleaning and washing. On one occasion, she had escaped from the house and was found wandering aimlessly in the neighborhood. At the time, it was also reported that she had been improperly dressed, in a manner that is deemed to be socially immodest in a rural, conservative society.

A protracted interview with her husband and siblings indicated that her distress tended to occur at the onset of menstrual flow. Her symptoms continued until the end of menstrual flow. She had a patchy memory of her state of clouded sensorium. She stated that she often felt tired and dysphoric during a certain time of the month which she attributed to stress at work.

Premorbid, there was no indication that BB had experienced any adverse life events during her childhood. She completed 12 years of education and later enrolled herself in a higher education institution to distinguish herself as a teacher. She has 11 siblings with no evidence of mental illness in the family. Her menses began at age 12.

BB denied having consumed any mind-altering substance including tobacco or its rejuvenated forms or alcohol. A routine urine drug screening did not reveal the presence of any illicit substances in her system.

Physical examination was unremarkable and her medical workup—including hormonal study, brain CT scan, and EEG—was inconclusive. The clinical team suggested that the respondent displayed manic episodes with psychotic symptoms or according to ICD-10, the client’s distress might be parallel to a manic episode, unspecified (F309)/brief psychotic disorder (F24). CIDI did not indicate the presence of typical manic (F30), Bipolar affective disorder (F31), schizophrenia or other psychotic disorders (F20, F22, F23, or F25).

Her distress subsided immediately upon admission and she was discharged with antipsychotic olanzapine (5 mg) for which she was compliant. Following up a week later, she was deemed suitable for protracted psychometric evaluation. Her intellectual and neuropsychological functioning are depicted in Fig. 1. BB scored 21 on the ***Calgary Depression Scale for Schizophrenia*** [17] which implied the presence of moderate depressive symptoms. Six months later, still compliant to prescribed medication (olanzapine 5 mg), she reported two episodes of relapse but with less intense symptoms. The two episodes of relapse once again coincided with menstruation and subsided with the end of her menstrual flow.

### Case report 3 (CC): premenstrual psychosis

CC, a 25-year-old, had been referred to this unit after she developed erratic behavior while traveling abroad. As detailed in the summary that was brought to our attention, she was sectioned under the mental health act and upon returning to her premorbid state, she was allowed to fly back to Oman. In the discharged summary, she was given a tentative diagnosis of an acute psychotic/manic episode. She responded when prescribed with Olanzapine (10 mg BD).

Upon arrival in Oman, she sought consultation with the present unit. The accompanying family member informed the clinical team of her distress while traveling abroad, of which CC has minimal recollection. The family informed us that she often experienced uneasiness with others and exhibited strongly inappropriate emotion and culturally devalued conduct in the last 5 years. They noted that the distress occurred periodically (approximately every 29 days) with abrupt onset during a full moon. In traditional Omani society, certain lunar cycles are thought to trigger bad omens and malevolent spirits. Her symptoms were deemed manageable by the family since they appeared to dissipate with lunar changes. Further exploration of her changed self and conduct appeared to occur during the second half of her menstrual cycle and end at the onset of menstrual bleeding.

Premorbid, her life during childhood was uneventful. She excelled in her education, graduated with a university degree and was on the lookout for a job. CC denied having consumed any mind-altering substances including tobacco or its rejuvenated forms and alcohol. A routine urine drug screening did not reveal the presence of any illicit drugs in her system. Physical examination was unremarkable and her medical workup—including hormonal study, brain CT scan, and EEG—was inconclusive.

CC and her family were offered the option of continuing with the same medication she was prescribed abroad. CC and her family refused the option under the pretext that the medicine (Olanzapine) left her feeling drowsy, constipated and with an insatiable appetite. The attending team labeled her of having something akin to a manic episode with psychotic symptoms. Using the ICD-10, she was registered in her medical records as having *Manic episode, unspecified (F309)*/brief psychotic disorder (F24) (Table 1). She was also subjected to intellectual and neuropsychological evaluation (see Fig. 1). CC scored 15 on the ***Calgary Depression Scale for Schizophrenia,*** a score suggesting the presence of mild depressive symptoms.

**Table 1:** Demographic and clinical variables of case-series with the association of attendees with menstrual psychosis at a tertiary care center in Oman

	Case #1 (AA)	Case # 2 (BB)	Case # 3 (CC)	Case # 4 (DD)
Education	Secondary school	University	University	Secondary
Age at time of testing	23	34	25	18
Marital status	Single	Married	Single	Married
Age of menses	13	12	13	12
Medication at time of assessment	Olanzapine (5 mg)	Olanzapine (5 mg)		olanzapine (5 mg)
CIDI/ICD-10	Manic episode, unspecified (F309)/brief psychotic disorder (F24)	Manic episode, unspecified (F309)/brief psychotic disorder (F24)	Manic episode, unspecified (F309)/brief psychotic disorder (F24)	*Manic episode, unspecified (F309)*/brief psychotic disorder (F24)
CDSS	23	21	15	9
Brockington’s subtypes of menstrual psychosis^a^	Premenstrual psychosis	Catamenial psychosis	Premenstrual psychosis	
Socio-cultural view	Spirit possession	Spirit possession	Spirit possession	Spirit possession

*CDSS* Calgary Depression Scale for Schizophrenia***:*** 0–9 = no depression; scores 10–15 = mild depression; scores 16–23 = moderate depression; scores > 24 = serious depression

^a^Brockington I. Menstrual psychosis. World Psychiatry. 2005 Feb; 4 (1): 9–17

*CIDI* Composite International Diagnostic Interview

*ICD* International Classification of Diseases

### Case report 4(DD): premenstrual psychosis

DD is an 18-year-old female: single and living in an urban-suburban area of the national capital, Muscat. She was brought to the hospital due to a sudden onset of distress. According to the accompanying family, she had been having episodes of disruptive behavior, impaired vegetative functioning and problems fulfilling activities of daily living. When she came out of prolonged sleep, she made irrelevant conversations and had episodes of incongruent crying. These episodes were followed by increased hyperactivity and agitation. She displayed tangentiality marked by exaggerated euphoria.

Due to the perceived temporality of her distress, the family devised mechanisms within the household to protect her well-being until the erratic behavior had dissipated. The accompanying sibling informed the clinical team that DD appeared to suddenly become disturbed around the second week of the lunar month. The family recalled that her distress occurred almost every month in the last 2 years. On previous occasions, the family invited a traditional healer who ‘diagnosed’ her as being possessed by the *jinn.* The fact that she had had little insight into her altered state led the family to believe that her altered state of consciousness represented all the hallmarks of spirit possession. During psychiatric consultation, the clinical team noted that her distress receded and this coincided with the onset of menstrual flow. It appeared, therefore, that her symptoms had persisted until the onset of menstrual flow.

DD was said to have met all her developmental milestones without difficulty. In Oman, individuals require 12 years to complete secondary education. She recalled that her menses began at 12 years old. She was deemed to have been very bright during her secondary school but upon reaching puberty, due to the aforementioned symptoms, she was only able to finish 9 years of formal education. Thereafter, as often is the case in Oman, she stayed within the confines of her extended and polygamous family. She has 5 siblings and no evidence of mental illness in the family.

A routine urine drug screening was not significant, and neither were her hormonal study, brain CT scan, and EEG. Semi-structured interview, CIDI, did not confirm that she had the core features of manic (F30) and Bipolar affective disorder (F31), schizophrenia and other psychotic disorders (F20, F22, F23, or F25). Rather, DD was deemed to be marked with something akin to manic episode with psychotic symptoms - Manic episode, unspecified (F309)/brief psychotic disorder (F24). The results of her psychometric evaluation are shown in Fig. 1, conducted when DD was stable.

Her disturbed behavior receded within 24 h of admission and she was discharged with antipsychotic olanzapine (5 mg) for which she was compliant**.** During the follow-up visit, she was stable and jovial and had already stopped her medication. The family and DD were given health education on how to adjust her life to accommodate for her predictable distress that occurred every month.

## Discussion

Studies from other populations have focused on the phenomenological indicators of menstrual psychosis and much (if not all) of the work on menstrual psychosis has been in the form of case studies taking a phenomenological approach [29]. To our knowledge, there are limited studies on cognitive and intellectual functioning among cases with menstrual psychosis using an idiographic approach [30]. As illness and distresses tend to be experienced in socio-cultural contexts, deciphering cultural idioms of distress is therefore imperative. As previous reports have only documented the clinical features of menstrual psychosis, this study has explored the intellectual and cognitive functioning of Omanis presenting with menstrual psychosis.

All reported cases from Oman appear to have adequate intellectual capacity marked with subtle and debilitating cognitive impairment in domains such as processing speed, executive functioning and, to some extent, episodic memory. Processing speed, executive functioning and episodic memory have been documented to constitute some of the core neuropsychological deficits among people with psychotic/manic episodes [31]. Interestingly, such cognitive impairments tend to persist even after the resolution of something akin to psychotic or manic symptoms [32] as observed in the present case series. In addition to intellectual and cognitive measures, the cases were evaluated for their affective range. Depressive symptoms were highly endorsed and, as is widely known, the presence of such symptoms has the potential to dent neuropsychological functioning [33].

Brockington [1] has also indicated that the difference in subtypes is due to time onset in relation to menstruation and duration. Brockington has defined menstrual psychosis as being different from the stages of reproductive life, i.e. those occurring with single episodes during menarche, puberty, amenorrhea, after childbirth and menopause. Brockington’s categorized subtypes are as follows: catamenial psychosis, paramenstrual psychosis, mid-cycle psychosis and epochal menstrual psychosis. A common feature of these subtypes is the cyclical presentation of clouded sensorium, shortness of episodes and abrupt onset of psychosis in a previously asymptomatic woman. This is followed by a total resolution of psychotic-like symptoms. Using the proposed classification of Brockington, the first (AA) and third case (CC) fit the description of premenstrual psychosis, i.e. psychotic or manic symptoms coinciding with the second half of the menstrual cycle. On the other hand, the second case (BB) appears to echo what Brockington postulated to be the hallmarks of catamenial psychosis.

One of the limitations of the present report is that tests of neuropsychological and intellectual functioning were conducted after symptoms of menstrual psychosis had receded; the rationale for this being that the clients were not accessible for psychometric evaluation during exacerbation of their distress. Future studies should, therefore, combine psychometric evaluation and functional brain scanning to shed light on the relationship between cognition and brain functioning. The second limitation of the present study is that some clients were still on medication while others were not. Medications are known to impact neuropsychological functioning [34]. Similarly, the clients were noted to have mild and moderate depressive symptoms. Thus, the observed neuropsychological pattern of reduced speed of processing and reduced executive functions could be linked to this particular mood state. A plethora of studies have suggested the presence of cognitive impairments in individuals with depressive symptoms [35]. Future studies should address this important confounder of neuropsychological functioning. Another limitation of this study, as highlighted by Brockington [1], was that to meet the required criteria, it is essential to adequately pinpoint the duration or precisely date the exacerbation of symptoms to confirm the diagnosis of menstrual psychosis, which was not currently done.

Although the pathophysiology of menstrual psychosis has remained elusive, speculations abound. Findings such as reduced dexamethasone suppression, disturbed circadian changes in cortisol levels and alteration in the Thyroid-Stimulating Hormone (TSH) response had led to the possibility of questioning hypothalamo-pituitary-adrenal system involvement [36]. Interestingly, these abnormalities are only detected during the active phase of menstrual psychosis and are abated between episodes. Another hypothesized contributory factor to menstrual psychosis is the role of progesterone [37]. Generally, estrogen is considered a neuroprotective steroid hormone and the reduction of estrogen during menstruation is considered to contribute to the exacerbation of psychosis.

With regard to nosology, Brockington [1] has argued that menstrual psychosis is a “morbid phenomenon related to bipolar disorder”. Shah et al. [5] postulated such a presentation as brief psychosis that rhythms around the menstrual cycle and resolves spontaneously and it, therefore, should be classified as psychotic *disorder not otherwise specified,* while Che [9] endorsed *transient* psychotic disorder according to the ICD-10 criteria. Due to the lack of direction in past literature, we agreed that the present case series from Oman would fit the criteria of a manic episode, unspecified (F309) or brief psychotic disorder (F24) since our hospital codes all diagnoses using the ICD-10. In a traditional society such as Oman, something akin to delirium, mania or mutism symptoms is likely to be attributed to spirit possession [37] or lunar cycles. The fact that most cases appear to be amnesic to their altered state further cements the ideas of an incarnating spirit being the main culprit and its monthly occurrence appears to echo the role of lunar cycles [38]. Spirit possession is one of the common “idioms of distress” or culturally salient indicators of distress in Oman. Altered states of mind here, are often attributed to an evil spirit of contemptuous envy, sorcery and/or spirit possession [39]. We have previously documented the magnitude of Omanis exhibiting something akin to the rapid dissolution of psychic/manic-like symptoms in Oman [40]. It had constituted 0.3% of those admitted to psychiatric services in a tertiary care center (*n* = 41,465) and 8.6% of the patients admitted to the psychiatry ward (*n* = 1294). It remains to be established how many of these patients were marked with menstrual psychosis.

While menstrual psychosis is recognized as being a rare clinical entity, under-recognition is likely to play a significant part. As exemplified by the cases in Oman, due to its brief duration associated with the menstrual cycle and full recovery later, menstrual psychosis might not be considered for scrutiny by biomedical services in a traditional society. In the future, concerted efforts are needed to further delineate where brief psychotic and manic-like conditions that are often presented with a culture-specific odium of distress should be featured in international psychiatric nomenclature. Since our hospital follows the ICD classification, the heuristic term, manic episode, unspecified (F309)/brief psychotic disorder (F24) was employed. But conceptually, unless further delineated, delirium, mania or mutism symptoms characterized by rapid onset and resolution with regular relapses might be also viewed as a brief psychotic disorder or premenstrual psychotic disorder. With the emerging interest in culture-bound/culture reactive syndromes [41], the possibility remains that these four cases from Oman might form part of a possessive syndrome that falls under the umbrella of culture-specific disorders in the ICD.

In thinking of management, Brockington [1] and Shah et al. [4] have suggested that antipsychotic treatment is usually ineffective and steroid hormones and clomiphene are more rational choices. Brockington [4] has stated that existing pharmacotherapies (such as antipsychotic medications) have the potential to ameliorate psychotic symptoms but such compounds are impervious to mitigating its recurrences as shown in the present cases. Instead, ‘unconventional compounds’ such as thyroid hormone and clomiphene have potential [4]. Given such background, Olanzapine was used as a symptomatic treatment due to the generally limited experience with menstrual psychosis and lack of evidence-based guidelines. Shah et al. [5] used a low dose of trifluoperazine as initial treatment for an Indian woman with menstrual psychosis which produced a good response. Moreover, Gerada & Reveley [42] used an antipsychotic to treat a 34-year old with rapid remission of the psychotic symptoms. Overall, the prevailing views suggest treatment with hormones or menstruation-suppressing agents as being possibly more effective. Estrogen, progesterone, androgen, and thyroxine have been successfully used in many cases as well [1]. Furthermore, clomiphene and danazole have also been used to mitigate the catatonic-like symptoms and cyclical recurrence of symptoms [11]. There have been no randomized control studies in this regard and most of the evidence has been derived from case reports [43].

## Conclusion

The clinical picture of this case-series highlights the uniqueness of menstrual psychosis as a distinct entity and its many manifestations appear to echo the subtypes highlighted by Brockington. This study is one of the first of its kind: documenting cognitive and intellectual functioning among cases with menstrual psychosis. While intellectually intact, the cases present cognitive impairments in domains that have been previously reported to be commonly affected among those with psychotic/manic disorders.

## Data Availability

The datasets used and/or analyzed during the current study is available from the corresponding author on reasonable request.

## Abbreviations

ICD-10: International Classification of Diseases and Health-Related Problems, Tenth Revision
DSM: Diagnostic and statistical manual of mental disorders
TSH: Thyroid-Stimulating Hormone
CT: Computerized Tomography
EEG: Electroencephalogram
CDSS: Calgary Depression Scale for Schizophrenia
RPM: Raven’s Progressive Matrices
IQ: Intelligence Quotient
